# The Place of PET to Assess New Therapeutic Effectiveness in Neurodegenerative Diseases

**DOI:** 10.1155/2018/7043578

**Published:** 2018-05-17

**Authors:** Anne-Claire Dupont, Bérenger Largeau, Denis Guilloteau, Maria Joao Santiago Ribeiro, Nicolas Arlicot

**Affiliations:** ^1^UMR 1253, iBrain, Université de Tours, Inserm, Tours, France; ^2^CHRU de Tours, Unité de Radiopharmacie, Tours, France; ^3^CHRU de Tours, Service de Médecine Nucléaire in vitro, Tours, France; ^4^INSERM CIC 1415, University Hospital, Tours, France; ^5^CHRU de Tours, Service de Médecine Nucléaire in vivo, Tours, France

## Abstract

In vivo exploration of neurodegenerative diseases by positron emission tomography (PET) imaging has matured over the last 20 years, using dedicated radiopharmaceuticals targeting cellular metabolism, neurotransmission, neuroinflammation, or abnormal protein aggregates (beta-amyloid and intracellular microtubule inclusions containing hyperphosphorylated tau). The ability of PET to characterize biological processes at the cellular and molecular levels enables early detection and identification of molecular mechanisms associated with disease progression, by providing accurate, reliable, and longitudinally reproducible quantitative biomarkers. Thus, PET imaging has become a relevant imaging method for monitoring response to therapy, approved as an outcome measure in bioclinical trials. The aim of this paper is to review and discuss the current inputs of PET in the assessment of therapeutic effectiveness in neurodegenerative diseases connected by common pathophysiological mechanisms, including Parkinson's disease, Huntington's disease, dementia, amyotrophic lateral sclerosis, multiple sclerosis, and also in psychiatric disorders. We also discuss opportunities for PET imaging to drive more personalized neuroprotective and therapeutic strategies, taking into account individual variability, within the growing framework of precision medicine.

## 1. Background

Neurodegenerative diseases (NDDs) are highly morbid hereditary and sporadic conditions characterized by progressive nervous system dysfunction and, ultimately, the loss of neurons. This heterogeneous group of disorders, including Alzheimer's disease (AD) and other dementias, Parkinson's disease (PD), multiple sclerosis (MS), amyotrophic lateral sclerosis (ALS), Huntington's disease (HD), is increasingly affecting the elderly worldwide, with a number of patients expected to double every 20 years [1]. Since these are progressive and irreversible disorders, early detection and differentiation of the disease are primordial for possible therapeutic intervention. Despite different initial clinical manifestations, many studies suggest that overlapping pathophysiologic processes may be involved in various forms of NDD, such as deposition of proteins with altered physicochemical properties in the human brain. Indeed, NDDs are thought to share a common pathogenesis mechanism, the aggregation and deposition of misfolded proteins not only in neurons but also in glial cells, which leads to progressive central nervous system impairments [2]. Thus, NDDs are classified according to current concepts of NDD based on clinical presentation, anatomical regions and cell types affected, and altered proteins involved in the pathogenetic process [3]. Basically, concerning correlation between anatomical involvement and NDD, it is well admitted that hippocampus, entorhinal cortex, or also limbic system are mainly involved in cognitive decline symptoms, whereas basal ganglia, thalamus, motor cortical areas, or cerebellar cortex are more involved in movement disorders. Amyloid-*β* (A*β*) and *τ*-protein aggregates in Alzheimer disease, as well as other forms of aggregates such as the *α*-synuclein aggregates (or Lewy bodies) found in Parkinson disease and dementia with Lewy bodies are among most proteins associated with the majority of NDDs. Concomitantly, microglial activation has also been linked with degenerative brain diseases by releasing proinflammatory cytokines including interleukin- (IL-) 1*β*, IL-6, and tumor necrosis factor- (TNF-) *α*, leading to neuronal damage and loss [4]. These common physiopathological processes suggest that these pathologies contribute to the development of other features of neurodegeneration such as neuronal and synaptic dysfunction in the central nervous system.

## 2. Current PET Imaging of Neurodegenerative Diseases

An early detection of the onset of NDD is pivotal as it can provide a chance for an early treatment that may prevent further progression of the disease. Over the past two decades, the traditional view of NDD, such as AD or PD, as purely clinical entity has been changed to one as a clinicobiological entity. A definite diagnosis has thus far been possible only by histopathologic postmortem assessment of brain tissue. Nevertheless, an important gap between the onset of symptoms and neuropathology in NDD is widely recognized. Hence, it has become increasingly possible to identify in vivo evidence of the specific neuropathology of NDD by use of validated biomarkers. Principal requirements for a good biomarker are preciseness, reliability, and capacity to distinguish healthy and pathological tissues. Among these, numerous neuroimaging biomarkers, being correlated with the NDD physiopathological process, have been introduced into the core diagnosis pathway. Positron emission tomography (PET) is a nuclear medicine imaging technique used to noninvasively assess various biological functions at the molecular level, by tracking a chemical compound of biological significance, called radiopharmaceutical, labeled with short-lived positron emitter radionuclide. In NDD, PET allows noninvasive evaluation of not only regional cerebral metabolism or perfusion but also the change of neurotransmission and presence of abnormal protein such as amyloid-*β*. ^18^F-FDG PET is a well-established radiopharmaceutical to measure regional glucose metabolism indicating neuronal function. In different forms of neurodegenerative dementias, specific patterns of neuronal dysfunction have been described [5]. Besides, dopamine transporter and vesicular monoamine transporter imaging are useful in the diagnosis and evaluation of Parkinson disease progression, providing information about the integrity of presynaptic striatal dopaminergic neurons. More recently, PET tracers for molecular imaging of A*β* have improved early diagnosis by targeting the amyloid deposition. Cholinergic and microglial imaging can be also useful in the early diagnosis of dementia and improve understanding of insights into pathophysiology of neurodegenerative diseases.

Therefore, the ability of molecular imaging to identify and quantify cerebral pathology has significant implications for early detection and differential diagnosis in NDD.

## 3. PET Neuroimaging Interest for Therapeutic Effectiveness Assessment

Molecular PET neuroimaging is a sensitive technique able to identify subtle molecular changes in the brain even before structural changes are present. Thus, one of the most important short-term roles of PET neuroimaging could be in the clinical evaluation and validation of new treatments of NDD, such as antiamyloid therapies in AD, which have entered in human trials (e.g., passive immunization, *γ*-secretase, and *β*-secretase inhibitors). Without a surrogate biomarker to assess the efficacy of these therapeutic agents on their intended central nervous system target, one cannot properly interpret the outcome of a therapeutic trial. All the more so as the assessment of clinical symptoms may therefore not represent an ideal tool for follow-up and therapy monitoring in NDD, it could have an important symptomatic overlap between NDD themselves. The ability of PET to not only provide spatial localization of metabolic changes but also to accurately and consistently quantify their distribution proved valuable for applications in assessment of drug effectiveness. Indeed, the great strength provided by functional and molecular PET approach allows visualizing numerous of the physiopathological pathways involved in NDD. The development of PET radioligands for the in vivo neuroimaging has been the focus of intense research efforts in recent years and most of the pathophysiologic processes involved in NDD mentioned above such as neuroinflammation, neurotransmission or misfolded protein aggregation, could to date be explored. Furthermore, the capacity to obtain quantitative information with PET tracer uptake in the brain could be relevant for the follow-up evaluation in therapy monitoring. The availability of plenty of PET tracers validated in humans (both on pharmacokinetic or dosimetry fields) provides exciting opportunities for the discovery, validation, and development of novel therapeutics in NDD. The new drug candidates may be radiolabeled in order to reflect, for instance, the biodistribution or the blood-brain barrier passage. But PET can especially be used to study the synthesis and release of neurotransmitters and the availability of neurotransmitter receptors. The growing epidemics of NDD such as AD, PD, or ALS have increased the need for new treatments, and their development is conditioned by first, the choice and the knowledge about the target and second, by the optimization of their validation in vivo. Known to be an important tool in both research and clinical care, PET neuroimaging approach in the therapeutic evaluation and optimization in NDD is discussed in this manuscript.

## 4. PET Imaging to Assess Therapeutic Effectiveness

### 4.1. Glucose Metabolism Imaging

Since its first application in humans in 1979 (Table 1) [13], ^18^F-FDG PET improves our understanding of many brain disorders. Indeed, its ability to measure local glucose consumption in various structures of the brain allows to detect alterations in local cerebral metabolism. ^18^F-FDG uptake by the cortical and subcortical structures in the brain has the advantage to provide valuable information before any morphological changes become discernible. Thus, ^18^F-FDG PET is a well-established tool to identify disease-specific cerebral metabolic brain patterns in several neurodegenerative brain diseases at an early disease stage. In AD, the most prevalent neurodegenerative cause of dementia [14], ^18^F-FDG is an effective modality for detecting functional brain changes since AD patients exhibit characteristic temporoparietal glucose hypometabolism. Furthermore, a correlation between the degree of hypometabolism and the severity of dementia has been reported during disease progression [15], in relation with neuronal cell loss and decreased synaptic activity. By assessing indirect functional effects of neurodegeneration, ^18^F-FDG can be useful for early diagnosis and the differential diagnosis between AD and other various types of dementia like dementia with Lewy bodies, frontotemporal lobe dementia, and vascular dementia. Thus, it is widely recognized that ^18^F-FDG holds a special place for the staging and assessment of AD. Unlike oncology field, where ^18^F-FDG is routinely used for treatment evaluation and follow-up, ^18^F-FDG has only been used sporadically in the past as a biomarker for predicting therapeutic response in AD. The first multicenter clinical trial in AD using ^18^F-FDG measuring brain glucose metabolism as the primary outcome has been described by Tzimopoulou et al. in 2010 [8]. Brain glucose metabolism was studied at baseline and at three later time points (1, 6, and 12 months) after 12 months treatment with the peroxisome proliferator-activated receptor (PPAR) gamma agonist rosiglitazone versus placebo in 80 mild-to-moderate AD patients. Rosiglitazone has been shown to ameliorate insulin resistance in patients with type II diabetes mellitus [16] and seems to improve cognition in AD in preliminary studies [17] but that effect could be limited to APOE4 subjects [18]. No statistically significant difference indicated that active treatment decreased the progression of decline in brain glucose metabolism over a one-year follow-up in the symptomatic stages of AD. Nevertheless, while failing to demonstrate an effect of rosiglitazone on neurodegeneration, these results are consistent with Phase III clinical trials using rosiglitazone in AD [19, 20], which conclude that PET imaging biomarker like ^18^F-FDG could provide good mechanistic tests for the evaluation of future therapeutic hypotheses. In 2016, in a safety and tolerability study of 6 months of pramipexole in 15 mild-to-moderate AD patients, Bennett et al. has used PET imaging to complete the study by examination of cognitive performance with ^18^F-FDG tracer. In this small single-arm, open-label study, there was no apparent effect of pramipexole because a 3–6% brain glucose uptake decrease has been observed during the 6-month follow-up, consistent with regions of reduced metabolism in AD patients without treatment [7]. Contrary to the minor interest of ^18^F-FDG in AD therapeutic assessment, a recent study has shown that apomorphine pump seems to be an interesting option for treating advanced PD patients in therapeutic impasse, thanks to a brain glucose metabolism study [9]. In 12 advanced PD patients, significant metabolic changes were observed, with overall increases in the right fusiform gyrus and hippocampus, alongside a decrease in the left middle frontal gyrus before and after 6 months of add-on apomorphine. Besides, consistent correlations between significant changes in clinical scores, mainly assessed according to UPDRS (Unified Parkinson's Disease Rating Scale) and MDRS (Mattis Dementia Rating Scale), and metabolism were established. In the same way, metabolic (by ^18^F-FDG-PET) and volumetric (by Magnetic Resonance Imaging-MRI) differences in the brain have been investigated to evaluate neuroprotective effects of riluzole in HD [21]. Riluzole interferes with glutamatergic neurotransmission, thereby reducing excitotoxicity and enhancing neurite formation in damaged motoneurons [22]. It also has been reported to inhibit voltage-gated sodium channels and to be neuroprotective by suppressing astrocytosis [23]. The 12 placebo-treated HD patients showed significantly greater proportional volume loss of grey matter and decrease in metabolic ^18^F-FDG uptake than the 11 HD patients treated with riluzole in all cortical areas (*p* < 0.05). Not only brain glucose metabolism was preserved in patients receiving riluzole, but also a correlation between the progressive metabolic consumption with worsening clinical scores (UHDRS-I, Unified Huntington Disease Rating Scale) in placebo group was reported. These findings corroborate that antiglutamatergic drugs like riluzole could represent a neuroprotective strategy in HD and that ^18^F-FDG-PET may be a valuable tool to assess brain markers of HD. Considered as a neurodegenerative or neurodevelopmental disorder, recent studies have shown the importance of treating schizophrenia, a chronic and severe mental disorder characterized by abnormal social behaviour and failure in assessing reality. Mostly, we distinguish positive (i.e. hallucinations, paranoid delusions, beliefs), negative (i.e. apathy, lack of emotion, and poor or nonexistent social functioning), and cognitive (disorganized thoughts, difficulty concentrating and/or following instructions, difficulty completing tasks, and memory problems) psychotic symptoms; and that is why many structural brain studies have correlated schizophrenia symptoms with reproducible structural brain abnormalities. For instance, progressive prefrontal grey matter atrophy is known to be more related to pronounced negative symptoms [24]. Cerebral metabolic studies with ^18^F-FDG have an interest to define brain regions associated with treatment-related improvement of symptoms in schizophrenic patients. Thus, increased relative metabolic rate has been observed in the frontal lobe in 30 psychotic patients treated with olanzapine versus no medication subjects [11]. Such a difference has not been observed in 17 patients previously exposed to antipsychotics [12]. Linking to the interest to treat schizophrenia as soon as possible, Yoshimuta et al. have examined the effects of olanzapine and identified brain regions associated with a positive response in neuroleptic-naive first-episode schizophrenic (FES) patients [10]. Glucose metabolism in responders was significantly increased after treatment in the left precentral gyrus, left postcentral gyrus, and left paracentral lobule and significantly decreased in the left hypothalamus. These observations added to the positive correlation between the changes in “Positive and Negative Syndrome Scale” (PANSS) scores and metabolic changes before and after treatment reinforce the beneficial action of olanzapine in FES patients.

### 4.2. Amyloid and Tau Imaging

Currently, the only FDA-approved AD drugs such as donepezil, galantamine, or memantine act partially on the symptoms of AD, without treating the underlying causes of the disease (Table 2). A worldwide quest is under way to find new treatments to stop, slow, or even prevent AD. Many of the new drugs in development aim at modifying the disease process itself, by impacting one or more of its hallmarks, like extracellular plaque deposits of the *β*-amyloid peptide (A*β*). For this purpose, interest of immunotherapy has grown during the last decade: antibodies are attractive drugs as they can be made highly specific for their target and often confer a lower risk of side effects in a vulnerable patient population during long-term treatment as compared with small-molecule anti-A*β* therapy. Thus, monoclonal antibodies have emerged to lower the beta-amyloid load in the brain, preventing the formation of plaques or even carrying excess beta-amyloid out of the brain. One of the earliest compounds evaluated for the treatment of AD was the bapineuzumab, a humanized N-terminal-specific anti-A*β* monoclonal antibody. Several PET studies measuring A*β* load have been performed for the clinical evaluation of this antibody, using radiopharmaceuticals developed from the chemical structure of histologic dyes. This noninvasive approach made it possible to track amyloid pathology longitudinally, following the disease progression.

In a study conducted by Rinne et al. in 2010 in 28 AD patients, the amyloid load was found to be reduced in the brains of patients treated with bapineuzumab as compared with placebo, as measured by binding of ^11^C-PIB to brain amyloid with PET [25]. In contrast, in a second Phase II study, bapineuzumab subcutaneous once monthly did not demonstrate a significant treatment difference over placebo on cerebral amyloid signal, assessed with ^18^F-florbetapir at one year [26]. Then, two Phase III trials of bapineuzumab in mild-to-moderate AD, supported by Janssen Alzheimer Immunotherapy Research & Development and Pfizer Inc. confirmed this result since bapineuzumab failed to reach the clinical endpoint in Phase III, namely, the overall negative clinical findings [27, 28]. The other humanized anti-A*β* monoclonal antibody that has been involved to large Phase III clinical trials is solanezumab, recognizing the central A*β*13–28 region [29]. Two large randomized double-blind controlled Phase III trials tested solanezumab as a potential treatment to slow the progression of mild-to-moderate AD, EXPEDITION 1 and EXPEDITION 2, with, respectively, 1012 and 1040 patients randomized to 400 mg of solanezumab or placebo every 4 weeks for 80 weeks [30]. Solanezumab failed to improve cognition or functional ability assessed with cognitive subscale of the Alzheimer's Disease Assessment Scale (ADAS-cog14) [31]. In both studies, a total of 169 patients and 97 in EXPEDITION 1 and 2, respectively, underwent baseline and follow-up ^18^F-florbetapir-PET scanning. The composite SUVR for the anterior and posterior right and left cingulate, plus right and left frontal, lateral temporal, and parietal regions, combined and normalized to the whole cerebellum, did no change significantly in the solanezumab group or the placebo group in either study. Many other anti-A*β* monoclonal antibodies are under development, and among them is the first fully human antibody, the gantenerumab that also binds specifically to A*β* plaques. The effect of up to 7 infusions of IV gantenerumab or placebo every 4 weeks on the A*β* amyloid load as measured by ^11^C-PiB has been studied in patients with mild-to-moderate AD in a preliminary PET study [32]. In 16 AD patients, the PET study has shown a dose-dependent reduction in brain A*β* plaques, but again no consistent treatment effects on cognitive measures were noted. Ongoing Phase III trials on gantenerumab on prodromal or mild stage of AD may clarify whether any reduction in brain A*β* deposits will successfully translate into clinical practice benefit at well-tolerated doses of gantenerumab [33]. Overall, to date, most of clinical trials trying to stop AD progression has led to reduce amyloid deposition but has little beneficial effect on cognitive improvement. Therefore, new approaches are being investigated, and a preliminary PET study has shown that benfotiamine significantly improved the cognitive abilities of 3 mild-to-moderate AD patients despite the progression of brain amyloid assessed by ^11^C-PIB [30]. Benfotiamine is a synthetic thiamine derivative preventing abnormal glucose metabolism via multiple pathways [34]. It is so demonstrated in this study that the alteration of cognitive capability is independent of brain amyloid accumulation, which is consistent with previous results showing that the reduction of brain amyloid accumulation by antibodies has little effect on the cognitive ability and disease progression of AD patients. It will be necessary to validate these results by randomized, double-blinded, placebo-controlled clinical trials.

As *β*-amyloid peptide, aggregates of hyperphosphorylated tau protein known as neurofibrillary tangles (NFTs) are one of the hallmarks of AD and related disorders, called tauopathies. Aggregates of tau are prominent targets for novel therapeutics as well as for biomarkers for diagnostic in vivo imaging. While immunotherapy targeting A*β* peptide gave poor results, tau-based immunotherapy clinical trials have recently emerged [35]. Promising results are expected from a new active vaccine, namely, AADvac1, targeting pathological tau protein in Alzheimer's disease [36]. In addition, the recent development of tau-specific PET tracers has allowed in vivo quantification of regional tau deposition and offers the opportunity to monitor the progression of tau pathology along with cognitive impairment. To our knowledge, any study with a tau PET tracer as a reliable outcome measure of drug efficacy assessment has been published yet. As explained by Okamura and Yanai [37], the methods to image analysis with tau PET tracer need to be optimized. Indeed, the variety of the different types of tau deposits is a crucial issue for the development in tau PET tracers. Recent data evidence the existence of off-target binding in areas of tau accumulation. Thus, a longitudinal observation of patients at baseline and post-selegiline (MAO-B inhibitor) ^18^F-THK5351 PET scans has tested the hypothesis that a reduction of MAO-B availability also reduces ^18^F-THK5351 uptake. In this study, Ng et al. reported that MAO-B was an ^18^F-THK5351 off-target binding site; hence, the interpretation of PET images is confounded by the high MAO-B availability [38].

With the increasing interest in antitau therapies, tau PET tracers will certainly be a tool to assess the therapeutic effects of these new drugs acting on tau load in the brain. For that purpose, new tau PET tracers (i.e., ^18^F-MK-6240 and ^18^F-AM-PBB3) have recently been reported to have less off-target binding than their predecessors [39].

### 4.3. Neuroinflammation Imaging

Initially discovered in Alzheimer's disease (AD), where activated microglia cells were found in postmortem nearby senile plaques (Table 3) [40], it is now clearly established that microglial activation and abnormal protein deposition take part in the process of neurodegenerative disorders such as AD, PD, ALS, and MS [4]. Thus, glial inflammation has heightened interest in the rapid discovery of neuroinflammation-targeted drugs [41]. Given the fact that anti-inflammatory drugs are able to suppress peripheral inflammation, many authors investigated their potential use for central nervous system (CNS) inflammation [42–44]. Nevertheless, only few clinical studies have evaluated, thanks to molecular imaging, and apart from clinical parameters, the ability of these drugs to reduce glial cell-propagated inflammation. In parallel, over the last 20 years, microglia PET imaging has successfully widened through the development of radiopharmaceuticals and the identification of several molecular targets of neuroinflammation. Among these targets, receptors including the translocator protein-18 kDa (TSPO) [45], cannabinoid receptor 2A [46], and adenosine receptor 2A [47, 48] and enzymes such as *β*-glucuronidase [49] have been targeted to evaluate the scope of microglia PET imaging in neurodegenerative disorders. To our knowledge, only TSPO PET imaging has been used to assess therapeutic efficacy in neurodegenerative disorders. Drugs evaluated in these studies include specific therapeutics which have already granted FDA licensure like interferon beta, [50] glatiramer acetate, [51] and fingolimod [52, 53] in MS, nonspecific drugs which exert anti-inflammatory effects, [54, 55] and new therapeutical class-targeting biochemical pathways involved in neurodegenerative disorder such as the hydrolysis of neuroprotective endocannabinoid [56] and oxidative stress [57]. Microglial activation plays a central role in maintaining the central chronic inflammation in MS [58]. MS is a chronic autoimmune disease of the CNS where the migration of myelin-reactive T-cells into the CNS is followed by microglial activation, recruitment of peripheral macrophages, and oligodendrocytes destruction [59]. Fingolimod blocks the egress of lymphocytes from secondary lymphoid tissues and thereby prevents their entry into the CNS [60]. In line with its mechanism of action, PET imaging showed that fingolimod reduced microglial activation [52, 53], especially in T2 lesion area [53]. Glatiramer acetate, a synthetic polypeptide resembling myelin basic protein, acts further downstream deceiving immune system and inducing immunomodulatory Th2 cells [61]. Ratchford et al. [51] provided proof of concept that microglia PET imaging with ^11^C-PK11195 could also be a tool to assess disease-modifying drugs for relapsing-remitting multiple sclerosis (RRMS) efficacity. Indeed, radiopharmaceutical binding potential per unit volume was statistically decreased in the whole brain after one year of glatiramer acetate. This result supported the in vitro evidence of its mechanism of action in which an inhibition of transformation to an activated microglia form could be responsible for therapeutic effects [61]. In other neurodegenerative disorders, TSPO PET studies have not achieved convincing results. Indeed, authors reported no significant difference [57] in microglial activation or a slight decrease [56] in TSPO density and sometimes an increase in TSPO tracer binding after therapeutic challenge [55].

### 4.4. Neurotransmission Imaging

During the past decades, numerous neurotransmitter systems have been identified and have been demonstrated to be directly involved in NDD. In vivo neuroimaging with PET using labeled ligands can visualize the various receptor and transporter systems and measure in quantitative terms their densities and binding and occupancy status (Table 4). The importance of PET in receptor-system-related drug research has increased tremendously in recent years.

One of the key monoamine neurotransmitters, the dopaminergic transmission plays a major role in neurological and psychiatric disorders such as PD, HD, and SCZ. Although mainly known to be involved in motor feature, dopamine is also involved in cognition and emotion. To investigate pre- and postsynaptic functions, PET tracers have been developed to measure dopamine synthesis and transport and postsynaptic receptors. For measuring dopamine synthesis, the most commonly used tracer is ^18^F-DOPA, whereas for dopamine transport, several radiolabeled tropane analogs have been developed. For postsynaptic dopamine receptors, divided on five subtypes of receptors, ^11^C-raclopride is the common tracer for D2/D3, whereas ^18^F-fallypride is mainly used for the exploration of D2 [62]. In clinical practice, it is well admitted that the extent of dopaminergic neuronal loss in the substantia nigra in PD patients is measured in vivo using ^18^F-DOPA considered as the gold-standard for monitoring the course of PD [63]. Unlike for other physiopathological pathways cited above as neuroinflammation or amyloid aggregation, dopamine molecular imaging has already widely been used for a long while to assess drug therapeutic effectiveness in PD essentially. Actually, it could be explained first by the fact that a treatment allowing restoration of dopamine neurotransmission is available for years for PD patients and second by direct dopaminergic transmission radiotracers availability. In 1990s, PET studies with ^11^C-raclopride have demonstrated the downregulation of the striatal D_2_ receptor binding in PD related to long-term treatment. Indeed, compared to the baseline, ^11^C-raclopride binding was significantly decreased in the putamen and caudate nucleus in PD patients treated for 3–5 years with L-DOPA or lisuride, whereas no change was observed in D2 density 3–4 months posttreatment [64, 65]. More recently, ^11^C-raclopride PET studies have evaluated the relationship between clinical improvement following a single oral dose of levodopa and drug-induced synaptic dopamine increases. A significant increase of striatal DA in both caudate and putamen after levodopa administration was correlated with the improvement of rigidity and bradykinesia, whereas tremor and axial symptoms are not found to be related to this striatal synaptic dopamine level [66]. This last study indicates that pathways other than nigrostriatal pathway may be implicated in the pathogenesis of parkinsonian tremor and axial features and so other treatments are expected. In parallel, dopaminergic system molecular imaging has broadly been investigated in levodopa-induced dyskinesias (LID) field [67]. LIDs are associated with increased and fluctuating synaptic dopamine levels following levodopa administration [68]. Finally, dopamine in vivo imaging has extensively been used to assess safety after neural transplantation [69–73].

Finally, PET imaging in clinical transplantation trials can provide additional valuable information alongside clinical observations. Although being used in almost ever case in PD study, dopamine transmission in vivo imaging has measured brain MAO-B inhibition in patients with AD and elderly controls after oral administration of sembragiline [74]. In order to assist in dose selection of the Phase 2 sembragiline study in patients with moderate AD, Sturm et al. had to determine the relationship between exposure to Sembragiline and the inhibition of MAO-B enzyme activity in the brain, thanks to ^11^C-L-Deprenyl-D2.

In addition to the dopamine neurotransmission, radioligands have been developed to target cholinergic, serotoninergic, or gabaergic transmissions. Thanks to two cholinergic system tracers (^11^C-PMP and ^11^C-nicotine), we can see the acetylcholinesterase inhibition by the galantamine up to 12 months in 18 mild AD patients [75]. The inhibitory *γ*-aminobutyric acid (GABA) is known to be involved in a number of neuropsychiatric disorders including schizophrenia, and that is why, the tiagabine effect, which increases synaptic GABA, has been investigated with ^11^C-Ro15-4513 in 12 male participants to show its potential involvement in schizophrenia. Tiagabine produced significant reductions in hippocampal, parahippocampal, amygdala, and anterior cingulate synaptic tracer binding, suggesting that acute increases in endogenous synaptic GABA are detectable in the living human brain using ^11^C-Ro15-4513 PET [76]. Then, an in vivo impairment in GABA transmission in schizophrenia has been recently demonstrated with ^11^C-flumazenil after administration of tiagabine, in 17 off-medication patients with schizophrenia and 22 healthy comparison subjects [77]. Finally, anectodal evidence suggests that serotonine PET imaging could also be interesting to assess buspirone efficacy in PD [73].

## 5. Discussion

As PET has become increasingly available and as the range of available brain radioligands continues to expand, the use of PET neuroimaging has increased in drug development assessment in recent years. To date, the neurotransmitter system which has been most widely studied in humans is the dopaminergic system, mainly explored in movement disorders notably, thanks to ^18^F-DOPA or even ^11^C-raclopride. These dopaminergic relevant biomarkers allowed to improve our knowledge about why PD patients develop daily fluctuations in mobility and troublesome involuntary movements after several years of dopamine replacement therapy. In vivo dopamine imaging could also help to improve PD patient selection in future clinical trials by selecting those with better predicted outcomes. Another physiopathological approach has surged recently with the development of PET amyloid radioligands. Developed and approved for clinical use as important diagnosis and prognostic biomarkers for AD or mild cognitive impairment (MCI) patients, amyloid tracers are also being used to evaluate therapeutic interventions. Thus far, clinical trials of promising treatment for AD have failed to significantly stop the disease progression [78]. Surprisingly, while almost all research effort has been focused on antiamyloid therapy for AD, a recent PET study shows that the alteration of cognitive capability is independent of brain amyloid accumulation, and thus, also other physiopathological ways have to be explored to try to reduce AD progression [30].

Thus, it may be of interest to provide perspectives on new targets for which PET tracers are currently under development and which are also considered relevant for therapeutic management of NDD:

- Purinergic ion channel receptors, and especially P2X7 receptor (P2X7R), are known to be overexpressed in activated microglia in animal models of neurodegenerative diseases, such as AD [79], ALS [80], or HD [81], and might be a promising target to assess therapeutics, especially since the GSK1482160, a strong P2X7R antagonist, has been evaluated in Phase 1 clinical study by GSK company [82]. Labeled with carbon-11, ^11^C-GSK1482160 is a promising radioligand for neuroinflammation PET imaging, and one would think that this P2X7R antagonist could be an excellent candidate for a theranostic approach.
- Regarding the protein accumulation, three major types of aggregated hyperphosphorylated proteins (amyloid-beta, tau, and alpha-synuclein (*α*-syn)) are involved in the pathogenesis of a variety of neurodegenerative diseases, referred to as proteinopathies. Indeed, PD, Lewy body dementia, and multisystem atrophy are part of a family called synucleinopathies. We have described the importance of amyloid-beta and tau tracers and the criticality of developing selective PET tracers for each type of aggregate above, in order to assess their relative contribution in pathogenesis. *α*-Syn appears undoubtedly to be an excellent target for PET radiotracer development for PD and other synucleinopathies. *α*-Synuclein inclusions (Lewy bodies) appear before dopaminergic changes, (i.e., premotor PD) so imaging *α*-syn could better predict premotor PD [83]. While success in the development of selective *α*-syn PET imaging agents has not been realized yet, *α*-syn radiotracer could be a potentially useful surrogate marker in clinical trials. Work is ongoing in multiple laboratories throughout the world, and AC Immune and Biogen companies have identified two lead compounds designed to selectively bind to *α*-syn aggregates.
- As mentioned earlier, numerous neurotransmitter systems have been identified and allowed to assess therapeutics. Among them, the cholinergic system could be of interest for the follow-up of NDD and their treatment. Degeneration of cholinergic neurons is well described in pathophysiology of AD and is associated in several reports with a significant loss of *α*7 nicotinic acetylcholine receptor (*α*7-nAChRs) in the cortex and hippocampus of patients. *α*7-nAChR mediates various brain functions and represents an important target for drug discovery. In clinical trials with selective *α*7 agonists, activation of the receptor improved cognitive performance in patients with schizophrenia [84]. The recently developed ^18^F-ASEM, a highly *α*7-nAChR specific and selective radiotracer for brain PET, opens new horizons for studying *α*7-nAChRs in the living human brain.

Finally, PET imaging in NDD therapeutic development assessment can lead to (i) the study of the role and density of receptor involved, (ii) the study of the mechanism of action of therapeutic drug, and (iii) the optimization of new treatment development by reducing costs and the time required for new drug development.

Nevertheless, the expansion of PET imaging as a reliable biomarker for in vivo treatment evaluation faces the critical lack of effective treatment for NDD patients, especially for AD. Concurrently, new potential applications of these radiotracers initially developed for central application have shown their interest in the field of personalized medicine in numerous peripheral diseases, including cancer. ^18^F-DOPA illustrates this concept since it is a well-known excellent tracer for imaging neuroendocrine tumors (NETs), including pheochromocytoma, extraadrenal paraganglioma, medullary thyroid carcinoma, gastro-entero-pancreatic (GEP), or NE tumors (reviewed in [85]). In NET, ^18^F-DOPA may be particularly useful in patients with negative ^68^Ga-somatostatin analogs. More recently, TSPO PET imaging has been shown to assume a promising involvement in the development of diagnostic strategies in cancer. More recently, TSPO has been introduced as a possible molecular target for peripheral sterile inflammatory diseases PET imaging, making this protein a potential biomarker with the aim of addressing disease heterogeneity, assisting in patient stratification, and contributing to predicting treatment response [86–88]. Finally, amyloid tracers such as ^18^F-florbetapir or ^11^C-PIB may be promising PET radiotracers for imaging amyloid deposit in cardiac amyloidosis [89, 90], considering that they also exhibit specific affinity for myocardial amyloid fibers.

It would be consider that this field of investigation will grow up in the context of personalized and/or stratified medicine.

## 6. Conclusion

This paper has reviewed findings from PET neuroimaging studies which have contributed to assess efficacy of drugs in NDD. In the last decades, molecular imaging with PET led to the progress in the development of new drugs, thanks to multiple molecular probes imaging biological, functional, and pathological conditions of NDD. Brain PET imaging allows a multiple approach of the disease by assessing several physiopathological pathways like neuroinflammation, neurotransmission, or protein aggregation in the disease. This multiple approach allows to assess drug efficacy from different perspectives and forms the link between clinical and physiopathological conditions. Complementary to the recent concept called “theranostics” referring to the use of molecular targeting vectors labeled either with diagnostic or therapeutic radionuclides for diagnosis and therapy, respectively, brain PET imaging seems to be a relevant and attractive tool in SNC drug development that could help in therapeutic decision-making within the growing framework of precision medicine.

## Figures and Tables

**Table 1:** Glucose metabolism imaging approach to assess therapeutic effectiveness.

Disorder	Physiopathological approach	Radioligand	Population	Therapeutical class	Main findings	References
HD	Glucose metabolism	^18^F-FDG	11 riluzole-treated HD patients versus 12 untreated HD patients	Riluzole (benzothiazole)	Placebo-treated patients showed significantly greater proportional volume loss of grey matter and decrease in metabolic FDG uptake than patients treated with riluzole in all cortical areas (*p* < 0.05).	Squitieri et al. [6]

AD	Glucose metabolism	^18^F-FDG	6 months treatment pramipexole 20 mild-to-moderate AD, 15 completed all visits	Pramipexole (D2-family dopamine receptor agonist)	Broad regions where glucose uptake decreased suggesting a relative decrease in metabolism (consistent with regions of reduced metabolism in individuals with AD). Nonapparent effect of r-pramipexole on brain regional glucose uptake.	Benett et al. [7]
	Glucose metabolism	^18^F-FDG	12 months treatment rosiglitazone versus placebo in 80 mild-to-moderate AD patients	Rosiglitazone (PPAR agonist)	Rosiglitazone is associated with an early increase in whole brain glucose metabolism but not with any biological or clinical evidence for slowing progression over a 1 year follow up in the symptomatic stages of AD.	Tzimopoulou et al. [8]

PD	Glucose metabolism	^18^F-FDG	12 patients with advanced PD were assessed before and after 6 months of add-on apomorphine	Apomorphine	Significant metabolic changes were observed, with overall increases in the right fusiform gyrus and hippocampus, alongside a decrease in the left middle frontal gyrus. Consistent correlations between significant changes in clinical scores and metabolism were established.	Auffret et al. [9]

SCZ	Glucose metabolism	^18^F-FDG	18 neuroleptic-naïve first-episode schizophrenic patients	Olanzapine	Glucose metabolism in responders was significantly increased after treatment in the left precentral gyrus, left postcentral gyrus, and left paracentral lobule and significantly decreased in the left hypothalamus.	Yoshimuta et al. [10]
	Glucose metabolism	^18^F-FDG	30 never-previously medicated psychotic adolescents (ages 13–20)	Olanzapine or haloperidol	Individuals treated with olanzapine showed increased relative metabolic rates in the frontal lobe more than the occipital lobe while patients treated with haloperidol failed to show increase in frontal metabolic rates and did not show an anteroposterior gradient in medication response.	Buchsbaum et al. [11]
	Glucose metabolism	^18^F-FDG	17 schizophrenic patients previously treated with antipsychotics	Olanzapine	No significant regional metabolic changes were related to previous treatment with classical neuroleptics.	Molina et al. [12]

AD, Alzheimer disease; FDG, fluorodeoxyglucose; HD, Huntington disease; PD, Parkinson disease; PPAR, peroxisome proliferator-activated receptor; SCZ, schizophrenia.

**Table 2:** Amyloid imaging approach to assess therapeutic effectiveness.

Disorder	Physiopathological approach	Radioligand	Population	Therapeutical class	Main findings	References
AD	Amyloid	^18^F-florbetapir	12 month study to determine long-term effects of monthly SC injections on 146 amyloid positive patients with mild to moderate AD (36 placebo versus 110 bapineuzumab 2 mg, 7 mg, 20 mg)	Bapineuzumab immunotherapy	Bapineuzumab did not demonstrate significant difference over placebo on GCA of 5 SUVR ROI: anterior cingulate, frontal cortex, lateral temporal cortex, parietal cortex, posterior cingulate/precuneus).	Brody et al. [26]
					Subgroup analysis based on disease severity: change in SUVR significant only in 7 mg/month group in patient with mild AD.	
	Amyloid	^11^C-PiB	78 weeks study on 28 patients with mild to moderate AD (8 placebo versus 20 Bapineuzumab IV 0.5 mg/kg, 10 mg/kg, 2.0 mg/kg)	Bapineuzumab immunotherapy	Estimated difference on cerebral retention ratio is significant between Bapineuzumab and placebo group.	Rinne et al. [25]
			(7 placebo and 19 bapineuzumab included in PiB PET analysis)		¹¹C-PiB PET seems to be useful in assessing the effects of potential AD treatments on cortical fibrillar amyloid-*β* load in vivo.	
	Amyloid	^11^C-PiB	Patients with mild to moderate AD	Bapineuzumab immunotherapy	Change from baseline to week 78 for GCA SUVR was not statistically significant versus placebo in either study.	Vandenberghe et al. [27]
			2 substudies: ApoEε4 carriers (24 placebo versus 32 bapineuzumab IV 0.5 mg/kg) ApoEε4 non carriers (13 placebo versus 17 bapineuzumab IV 0,5 mg/kg)		No significant treatment differences were seen in amyloid burden on PiB-PET.	
	Amyloid	^11^C-PiB	Patients with mild to moderate AD	Bapineuzumab immunotherapy	Significant differences in mean SUVR were only observed between bapineuzumab and placebo in the ApoE ε4 carrier study.	Salloway et al. [28]
			PiB PET substudy: ApoE ε4 carriers: 75 Bapineuzumab IV versus 40 placebo; ApoE ε4 non-carriers: 22 Bapineuzumab IV versus 15 placebo			
	Amyloid	^18^F-florbetapir	Patients with mild-to-moderate AD receiving placebo or solanezumab IV 4 weeks for 18 months	Solanezumab immunotherapy	The composite SUVR for the anterior and posterior right and left cingulate, plus right and left frontal, lateral temporal, and parietal regions, combined and normalized to the whole cerebellum, did no change significantly in the solanezumab group or the placebo group in either study.	Doody et al. [29]
			Combining the 2 substudies, a total of 266 patients underwent PET examination at baseline and week 80 or early termination			

**Table 3:** Neuroinflammation imaging approach to assess therapeutic effectiveness.

Disorder	Physiopathological approach	Radioligand	Population	Therapeutical class	Main findings	References
AD	Neuroinflammation TSPO	^18^F-GE180	Preclinical: transgenic APdE9 mice were treated with JZL184 (*n*=7) or with vehicle (*n*=5)	Monoacylglycerol lipase inhibitor: JZL184 40 mg/kg/d	In JZL184-treated mice, there was a very slight decreasing trend in tracer uptake in multiple brains areas compared to vehicle-treated APdE9 mice.	Pihlaja et al. [56]

MS	Neuroinflammation TSPO	^18^F-GE180	Preclinical: chronic focal EAE-like lesions were induced in rats via stereotactic intrastriatal injection of BCG and subsequent activation using an intradermal injection of BCG	Selective immunosuppressant (S1P receptor antagonist): fingolimod 0.3 mg/kg/d	Treatment with fingolimod for 28 days resulted in a clear reduction in the binding of trace when compared with vehicle-treated animals.	Airas et al. [52]
			Animals were then treated for 28 days with either fingolimod (*n*=5) or vehicle (*n*=3)		Quantification of the binding of the radiotracer revealed a significant reduction in the BP_ND_ of ^18^F-GE180 after treatment with fingolimod.	
	Neuroinflammation TSPO	^11^C-PK11195	Clinical: 9 drug-naïve RRMS patients were scanned at baseline before initiating GA and again after 1 year of GA treatment	Immunomodulatory: GA 20 mg/d	Significantly decreased tracer BP_ND_ per unit volume 3.17% in whole brain between baseline and 1 year and especially in supratentorial brain, infratentorial brain, cerebral white matter, cortical grey matter, thalamus and putamen.	Ratchord et al. [51]
	Neuroinflammation TSPO	^18^F-FEDAA1106	Clinical: RRMS patients in acute relapse: 6 drug-naïve patients and 3 patients on interferon beta therapy versus 5 HC	Immunomodulatory: interferon beta	No significant difference of tracer BP_ND_ or V_T_ between the three groups: MS without treatment, MS with interferon beta therapy, and normal control.	Takano et al. [50]
	Neuroinflammation TSPO	^11^C-PK11195	Clinical: 10 RRMS patients were scanned at baseline before initiating fingolimod and again after 6 months of treatment	Selective immunosuppressant (S1P receptor antagonist): fingolimod	Tracer binding was reduced (−12.3%) in the T2 lesion area after 6 months of fingolimod treatment, but not in the areas of NAWM or grey matter.	Sucksdorff et al. [53]
			7 patients were scanned 2 months following the treatment initiation		5/7 patients showed a slight increase tracer DVR in NAWM during the first 2 months of fingolimod treatment.	
					6/7 patients showed a slight increase in cortical gray matter after 2 months.	

MSA	Neuroinflammation TSPO	^11^C-PK11195	Clinical: 8 MSA-P patients: 3 with minocycline versus 5 in placebo arm	Tetracycline with anti-inflammatory effects: minocycline 200 mg/d	Compared to baseline, tracer BP_ND_ decreased in caudate nucleus, thalamus, midbrain and cerebellum for 2/3 treated patients after 24 weeks of minocycline.	Dodel et al. [54]
			Two groups were followed up for 6 months		In placebo group, tracer BP_ND_ is increased in most regions after 6 months.	

PD	Neuroinflammation TSPO	^11^C-PK11195	Clinical: 5 PD patients were scanned before and after one month of celecoxib	COX-2 inhibitor: celecoxib 100 mg/d	Tracer binding potential and distribution volume after celecoxib treatment were slightly higher.	Bartels et al. [55]
	Neuroinflammation TSPO	^11^C-PBR28	Clinical: 24 PD patients: 18 with AZD3241 versus 6 in placebo arm	Myeloperoxidase inhibitor: AZD3241 600 mg/12 h	There was no significant difference in changes of V_T_ between the treatment groups.	Jucaite et al. [57]
			16 PD patients in AZD3241 arm were followed-up for 8 weeks		AZD3241 significantly reduced V_T_ in regions of the nigrostriatal pathway compared to baseline V_T_ values at 4 weeks and at 8 weeks.	

AD, Alzheimer's disease; BCG, bacillus Calmette-Guérin; BP_ND_, binding potential; DVR, distribution volume ratio; EAE, experimental autoimmune encephalitis; GA, glatiramer acetate; MS, multiple sclerosis; MSA, multiple system atrophy, NAWM, normal appearing white matter; PD, Parkinson's disease; RRMS, relapsing-remitting multiple sclerosis; S1P, sphingosine 1-phosphate; V_T_, total distribution volume.

**Table 4:** Neurotransmission imaging approach to assess therapeutic effectiveness.

Disorder	Physiopathological approach	Radioligand	Population	Therapeutical class	Main findings	References
PD	Dopamine neurotransmission	^11^C-raclopride	18 previously untreated PD patients and 14 healthy volunteer subjects	Levodopa/lisuride	3 to 4 months' oral therapy with LD or lisuride does not change striatal dopamine D2-receptor density in PD patients.	Antonini et al. [64]
	Dopamine neurotransmission	^11^C-raclopride	9 patients with PD at an early drug-naive stage and 3–5 years later and 10 healthy controls in the same age range	Levodopa/lisuride	After 3–5 years, binding was significantly reduced in the putamen (*p* < 0.03) and caudate nucleus (*p* < 0.03) compared with baseline. These results indicate long-term downregulation of striatal dopamine D2 receptor binding in PD.	Antonini et al. [65]
	Dopamine neurotransmission	^11^C-raclopride	16 advanced PD patients	Levodopa	Following LD, mean caudate and putamen ^11^C-raclopride BPs were significantly lower versus baseline, consistent with increased synaptic DA.	Pavese et al. [66]
					A two-scan RAC PET study has reported, in advanced PD cases that, improvement in bradykinesia and rigidity scores following oral DA medication administration were significantly correlated with reductions in RAC binding suggesting an effect of increased DA on the striatal D2 receptors.	
	Dopamine neurotransmission	^18^F-6-fluorodopa	67 patients with IP, 52 with fluctuations and 15 with a stable response to LD	Levodopa	A 28% decrease in presynaptic terminal function in the putamen of PD patients with a fluctuating response to LD compared to the stable responders.	de la Fuente-Fernández et al. [68]
	Dopamine neurotransmission	^18^F-6-fluorodopa	9 patients with PD followed for 10 to 72 months after human embryonic mesencephalic tissue	Human embryonic mesencephalic tissue	^18^F-6-DOPA uptake increases in the striatum following the transplantation.	Wenning et al. [67]
	Dopamine neurotransmission	^18^F-6-fluorodopa	40 patients with PD	Transplantation of human embryonic dopamine neurons	Among younger patients (60 years old or younger), standardized tests of PD revealed significant improvement in the transplantation group as compared with the sham-surgery group when patients were tested in the morning before receiving medication.	Freed et al. [70]
	Dopamine neurotransmission	^18^F-6-fluorodopa	34 patients with advanced PD	Fetal nigral transplantation	Striatal ^18^F-6-DOPA uptake was significantly increased after transplantation in both groups and robust survival of dopamine neurons was observed at postmortem examination.	Olanow et al. [71]
AD	MAO-B inhibition dopamine neurotransmission	^11^C-L-deprenyl-D2	10 AD patients versus 6 elderly control subjects	Sembragiline MAO-B inhibitor	This PET study confirmed that daily treatment of at least 1 mg sembragiline resulted in near-maximal inhibition of brain MAO-B enzyme in patients with AD.	Sturm et al. [74]
	Acetylcholine neurotransmission	^11^C-PMP	18 subjects (12 galantamine versus 6 placebo) with mild to moderate AD (14 completed 12 months study)	Galantamine	In the galantamine group, there was significant inhibition of AChE activity in all four cortical regions (frontal, parietal, parietotemporal, temporal).	Kadir et al. [75]
		^11^C-nicotine			The placebo group did not show any significant inhibition compared with baseline.	
					A positive correlation was observed between changes in the average cortical ^11^C nicotine binding and plasma galantamine concentrations.	

PD	Serotonin neurotransmission	^11^C-DASB	2 PD patients who had shown recovery of motor function after intrastriatal fetal ventral mesencephalic tissue transplantation but experienced off-phase GIDs	Neural transplantation	It was found excessive serotoninergic innervation in the grafted putamen in one patient and putamen and caudate nucleus in second patient.	Politis et al. [73]
				Buspirone 5-HT_1A_ receptor agonist	Buspirone in low repeated dose markedly attenuated dyskinesia severity in both transplanted patients.	

SCZ	GABA neurotransmission	^11^C-flumazenil	17 off-medication SCZ patients versus 22 HC	Tiagabine	^11^C-flumazenil **V**_**T**_ was significantly increased across all cortical brain regions in the healthy comparison group but not in the schizophrenia group.	Frankle et al. [72]
				IGAT1		
	GABA neurotransmission	^11^C-Ro15-4513	12 healthy male completed the Tiagabine challenge study	Tiagabine	Tiagabine administration produced significant reductions in hippocampal, parahippocampal, amygdala, and anterior cingulate synaptic α1 ^11^C-Ro15-4513 binding, and a trend significance reduction in the nucleus accumbens.	Stokes et al. [76]
				IGAT1		

AChE, acetylcholinesterase; AD, Alzheimer disease; BP, binding potential; DA, dopamine; EC, elderly control; GIDs, graft-induced dyskinesia; HC, healthy comparison; MAO-B, monoamine oxydase type B; IP, idiopathic parkinsonism; LD, levodopa; PET, positron emission tomography; RAC, ^11^C-raclopride; SCZ, schizophrenia; V_T_, tissue distribution volume.
